# Oligo-metastatic neoPlasms from the gastro-intestinal tract: iDentIfiCaTIon of cliNical and molecular drivers: the PREDICTION study

**DOI:** 10.1186/s12885-023-11479-w

**Published:** 2023-10-19

**Authors:** Alessandro Ottaiano, Antonella De Luca, Mariachiara Santorsola, Giosuè Scognamiglio, Annabella Di Mauro, Paolo Chiodini, Matilde Lambiase, Alessandra Sacco, Antonella Petrillo, Vincenza Granata, Roberta Fusco, Edoardo Mercadante, Nicola Martucci, Giuseppe De Luca, Antonello La Rocca, Egidio Celentano, Anna Crispo, Piergiacomo Di Gennaro, Fabiana Tatangelo, Gerardo Ferrara, Francesco Izzo, Andrea Belli, Renato Patrone, Paolo Delrio, Daniela Rega, Silvia De Franciscis, Paolo Muto, Vincenzo Ravo, Rossella Di Franco, Valentina Borzillo, Sara Santagata, Giuseppina Rea, Daniela Castaldo, Ugo Pace, Gianfranco De Feo, Stefania Scala, Guglielmo Nasti, Nicola Normanno

**Affiliations:** 1 Istituto Nazionale Tumori, IRCCS “G. Pascale”, Napoli, 80131 Italy; 2 Section of Statistics, Department of Mental Health and Public Medicine, Università degli Studi della Campania Luigi Vanvitelli, Napoli, 80138 Italy

**Keywords:** Oligometastatic diseases, Colorectal cancer, Biomarkers, Genetics, Prognosis

## Abstract

**Background:**

Metastatic disease in tumors originating from the gastrointestinal tract can exhibit varying degrees of tumor burden at presentation. Some patients follow a less aggressive disease course, characterized by a limited number of metastatic sites, referred to as “oligo-metastatic disease” (OMD). The precise biological characteristics that define the oligometastatic behavior remain uncertain. In this study, we present a protocol designed to prospectively identify OMD, with the aim of proposing novel therapeutic approaches and monitoring strategies.

**Methods:**

The PREDICTION study is a monocentric, prospective, observational investigation. Enrolled patients will receive standard treatment, while translational activities will involve analysis of the tumor microenvironment and genomic profiling using immunohistochemistry and next-generation sequencing, respectively. The first primary objective (descriptive) is to determine the prevalence of biological characteristics in OMD derived from gastrointestinal tract neoplasms, including high genetic concordance between primary tumors and metastases, a significant infiltration of T lymphocytes, and the absence of clonal evolution favoring specific driver genes (*KRAS* and *PIK3CA*). The second co-primary objective (analytic) is to identify a prognostic score for true OMD, with a primary focus on metastatic colorectal cancer. The score will comprise genetic concordance (> 80%), high T-lymphocyte infiltration, and the absence of clonal evolution favoring driver genes. It is hypothesized that patients with true OMD (score 3+) will have a lower rate of progression/recurrence within one year (20%) compared to those with false OMD (80%). The endpoint of the co-primary objective is the rate of recurrence/progression at one year. Considering a reasonable probability (60%) of the three factors occurring simultaneously in true OMD (score 3+), using a significance level of α = 0.05 and a test power of 90%, the study requires a minimum enrollment of 32 patients.

**Discussion:**

Few studies have explored the precise genetic and biological features of OMD thus far. In clinical settings, the diagnosis of OMD is typically made retrospectively, as some patients who undergo intensive treatment for oligometastases develop polymetastatic diseases within a year, while others do not experience disease progression (true OMD). In the coming years, the identification of true OMD will allow us to employ more personalized and comprehensive strategies in cancer treatment.

**Trial registration:**

ClinicalTrials.gov ID NCT05806151.

## Background

Cancer spreading to distant organs, such as the lungs, liver, bones, and brain from primary tumors, remains the primary cause of cancer-related deaths. The extent of cancer spread is commonly assessed by factors including the number, size, and involvement of loco-regional lymph nodes, which are then used to guide clinical staging systems [1, 2]. However, metastatic disease in stage IV cancers can present with widely varying initial tumor burdens [3]. Patients may present with a highly metastatic pattern involving multiple sites, known as “poly-metastatic disease” (PMD), or with a less severe disease course involving fewer sites, referred to as “oligo-metastatic disease” (OMD) [4]. OMD manifests in 2–10% of patients across diverse cancer types, depending on the specific histological subtype. The term OMD is applied when the disease presents with no more than three lesions per organ and a total maximum size equal to 7 cm [5], or a primary tumor (active or resected) with ≤ 5 easily resectable lesions (with a size limit of 5 cm) [6], or controllable metastatic masses amenable to local approaches [7]. Oligo-metastatic patients generally have a favorable prognosis because the disease has a prolonged course that can be effectively controlled with local treatments (such as radiotherapy or radiofrequency ablation) and less aggressive systemic therapies. OMD is not merely an intermediate stage between localized, low-burden disease and diffuse metastases; it represents a distinct disease entity with specific biological and molecular features. The low “metastatic virulence” observed in OMD may reflect specific and dynamic aspects of tumor biology and/or host-tumor relationships [8]. Guckenberger et al. [9] interestingly redefined OMD in oncology as “induced” OMD (occurring in patients with a history of poly-metastatic cancer prior to the diagnosis of OMD) and “de novo” OMD (in patients without a history of poly-metastatic disease prior to the diagnosis of OMD), with the latter group having a favorable prognosis. However, the biological characteristics that uniquely identify oligometastatic behavior remain unknown, and the definition of a ‘true’ and good-prognosis OMD is necessarily retrospective, as many patients initially classified as oligometastatic eventually develop polymetastatic disease (PMD) within one year.

### Rationale

We have previously presented evidence regarding OMD in colorectal cancer [10–12]. Patients diagnosed with oligometastatic colorectal carcinoma generally exhibit a notably enhanced median survival rate, approximately 44.0 months, in comparison to those with PMD, where the median survival is 24.0 months [13]. Moreover, in two earlier studies, we established substantial genetic and immunological distinctions between OMD and PMD. Notably, we observed an elevated occurrence of the *ERBB2* p.Pro1170Ala variant in oligometastatic lung disease [14], and an absence of *PIK3CA* mutations in oligometastatic liver and lung disease [15]. Among the patients with OMD, 9 out of 11 (81.8%) showed reversion in *KRAS* mutation at metastatic sites, whereas only 1 out of 52 (1.9%) with progressive PMD exhibited *KRAS* mutation reversal, with the majority acquiring *KRAS* mutations [16]. Consistently, tumor DNA sequencing results revealed a high level of genetic concordance in patients with OMD, with genetic coding variants showing over 80% concordance between the primary tumor and corresponding oligo-metastases [10–12, 14, 15]. Additionally, we observed an increased infiltration of GRZB + lymphocytes, which are activated cytotoxic lymphocytes, in oligometastatic liver disease with a favorable prognosis, indicating complex immunological interactions within the tumor microenvironment [14, 15]. The primary objective of the PREDICTION study is to identify OMD from a biological and molecular perspective, aiming to propose innovative therapeutic and monitoring strategies.

### Study design

The PREDICTION study is a prospective, observational, and monocentric investigation. Patients enrolled in the study will receive standard treatment. Translational activities will include the analysis of the tumor microenvironment in all accessible lesions, as well as genomic profiling of samples from enrolled patients to assess genetic concordance between primary and metastatic tumors, and to examine the mutational evolution of key driver genes.

### Study population

The study population consists of patients affected by gastroenteric cancers (colon, stomach, biliary tract, exocrine glands of the digestive tract) with OMD. Oligometastatic patients present one to three lesions per organ with a maximum tumor diameter of less than 70 mm and no lesion with a diameter greater than 25 mm. The correct identification of oligometastatic patients for the study will be performed through radiological staging techniques such as Computed Tomography (CT) and/or Magnetic Resonance Imaging (MRI). It is important to differentiate between two clinical presentations in OMD: those presenting with “oligo-recurrence” and those with “sync-oligometastatic” cases [17–19]. In the former, the primary tumor has been effectively controlled through a radical local approach, while in the latter, oligo-metastatic disease manifests synchronously with an active primary tumor. The PREDICTION study will include patients with both presentation types, contingent upon their having undergone radical treatment for all lesions.

### Primary objectives

This study has two main objectives. The first objective aims to determine the prevalence of specific biological characteristics in OMD arising from gastrointestinal tract neoplasms, including those in the colon, stomach, biliary tract, and exocrine glands of the digestive tract. These biological characteristics encompass: (1) high genetic concordance between primary tumors and metastases, (2) the presence of a high T lymphocyte infiltrate in the primary tumor and/or metastases, and (3) the absence of clonal evolution that favours specific driver genes. The primary endpoint of the first objective is to delineate the prevalence of these biological characteristics. Our center has the potential to annually recruit 11 new oligometastatic patients with colon tumors, 5 with stomach tumors, 3 with biliary tract tumors, and 3 with exocrine gland tumors of the digestive tract suffering from these pathologies.

The second co-primary objective aims to identify a prognostic score that identify true OMD. This score is tested in a single pathology, namely patients with metastatic colorectal cancer, which is the most common gastroenteric cancer associated with OMD. The score is based on the following characteristics, and possession of all characteristics (3+) constitutes the full score:

- Primitive/metastasis genetic concordance > 80% = 1 point;
- High T-lymphocyte infiltration - GRZB+ (> 10 cells/mm2) in the primary tumor and/or metastases (where tissue is available) = 1 point;
- Absence of clonal evolution favouring specific driver genes [absence of *KRAS* and/or *PIK3CA* mutations in metastases, even with a mutated primary (i.e., clonal evolution not favoring metastases)] = 1 point.

The hypothesis is that patients with true OMD (score 3+) have a significantly lower rate of progression at one year, defined as recurrence after radical surgery or progression based on RECIST v 1.1 criteria [20] since enrolment in the study, compared to those with false OMD who subsequently develop PMD. The primary endpoint for this objective is the rate of recurrence/progression within one year.

### Secondary objectives

- To describe the types of treatments received by patients with oligo-metastatic colon-rectal cancer;
- To measure the response evaluated according to Response Evaluation Criteria in Solid Tumors (RECIST) version 1.1;
- To measure the duration of the response, from the time the objective response is documented until progression;
- To evaluate progression-free survival (PFS) and overall survival (OS).
- To describe genetic and immunologic differences between “oligo-recurrence” and “sync-oligo-metastatic” cases.

### Study design and statistical analyses

The study aims to achieve its analytical/prognostic objective by enrolling a sufficient number of patients to demonstrate that “true” oligometastatic CRC (score 3+) have a significantly lower progression rate (defined as recurrence after radical surgery or progression according to RECIST v 1.1 criteria from the time of enrolment in the study) within 1 year compared to those with “false” OMD (score < 3+). The primary endpoint will be recurrence/progression within 1 year.

The sample size was determined using a two-sided test of difference between proportions to evaluate the statistical significance of the difference in recurrence within 1 year.

For this purpose, the following scenario was considered:

- A reasonable probability of the simultaneous occurrence of the 3 factors in true OMD (score 3+) of 60% [14, 15, 19];
- A recurrence rate of 20% for true OMD (score 3+) [11], and 80% for false OMD (score < 3+) [10–15, 19].

With a significance level of α = 0.05, a test power of 90%, and a Fisher exact test, the required number of patients to be enrolled is 32, to be recruited over an expected period of 3 years.

### Statistical methods

Patient characteristics will be presented as total numbers and percentages for categorical variables, and as the mean and standard deviation or median and interquartile range for continuous variables. Differences between groups will be assessed using the Chi-square test or Fisher’s exact test for categorical variables, and the Mann-Whitney U-test, Kruskal-Wallis test, t-test, or ANOVA for continuous variables. The choice of applying parametric or non-parametric tests will be based on the results of the Shapiro-Wilk normality test. The statistical tests will be two-tailed. For the evaluation of the first primary descriptive endpoint, summary statistics will be computed, and 95% confidence intervals will be calculated if necessary. To assess the second co-primary endpoint, Fisher’s exact test will be employed. In the context of survival endpoints, we will use the Kaplan-Meier estimator, and survival curves will be compared using the Log-rank test. The Cox proportional hazards regression model will be employed to calculate Hazard Ratios along with the appropriate 95% confidence intervals. Given the potential prognostic and biological differences between the sync-oligometastatic state, associated with a heightened risk of mortality compared to oligo-recurrence [21], a post-hoc analysis will be conducted to explore potential correlations between these two presentations and the genetic and immunological patterns previously delineated. This analysis will be presented upon the conclusion of the primary data analysis and within the primary description of the PREDICTION study.

### Patient selection

#### Inclusion criteria

- Diagnosis of gastroenteric cancers (colon, stomach, biliary tract, exocrine glands of the digestive tract);
- OMD: one to three lesions per organ with a maximum tumor diameter of less than 70 mm and no lesion with a diameter greater than 25 mm;
- Availability of FFPE (Formalin Fixed Paraffin Embedded) inclusions from resected primary tumor;
- Written informed consent.

#### Exclusion criteria

- Previous or concurrent malignant neoplasms;
- Presence of cerebral metastases;
- Refusal or inability to provide informed consent;
- Inability to guarantee follow-up.

### Screening procedures

The screening phase of the study will involve the evaluation of both inclusion and exclusion criteria. If deemed eligible, patients will be provided with information about the study objectives, treatment options determined by the oncologist following standard clinical practice, the necessity of conducting analyses on tissue samples collected during their treatment history, or in cases where metastatic tissue samples are unavailable, molecular characterization via liquid biopsy. Patients will also be informed about the procedures involved and the potential associated risks. To participate in the study, patients will be required to sign a specific informed consent form. The timing of study procedures is summarized in Table 1.

**Table 1:** Study procedures

Study assessments	Screening phase	Enrolment	At oligo-metastases treatment	Every 3 months	End of study	Follow-up
Informed consent	X					
Eligibility	X					
Pregnancy test	X					
Anamnesis	X					
Concurrent medications	X			X	X	X
Cardiologic evaluation	X			X		X
Height	X					
Weight	X			X	X	X
Clinical examination, ECOG PS, vital signs	X			X	X	X
Blood count and clinical biochemistry, CEA and CA19.9		X		X	X	X
Urinalysis		X			X	X
Creatinine clearance		X			X	X
Assessment of angiogenic factors		X				
Characterization of the tumor microenvironment of primary tumor		X				
Characterization of the tumor microenvironment of metastasis			X*			
Genetic assessment of the primary tumor		X				
Genetic assessment of the metastasis			X*			
Liquid biopsy			X**			
Total-body computed tomography with i.v. contrast (if contraindicated: abdomen MRI and chest computed tomography without i.v. contrast)	X			X	X	X
Adverse events evaluation				X	X	X
Progression				X	X	X
Survival				X	X	X

*After Surgical Treatment

**Oligo-metastatic patients not eligible for an upfront loco-regional treatment

### Treatments

The treatments will be determined by the referring oncologist in accordance with standard clinical practice. As a result, a patient’s decision to participate in this study will not impact treatment decisions in any way. If a patient is enrolled in an interventional therapeutic trial, the guidelines specified by the corresponding protocol will be followed.

## Methods

### Next-generation sequencing

Identifying genetic and immunological events that influence the clinical behavior and evolution of a neoplasm over time and space is a new frontier in research enabled by next-generation sequencing (NGS) techniques, which allow for the identification of various types of genomic alterations in a single experiment [22]. For the molecular characterization of tissue samples, genomic DNA will be extracted from formalin-fixed paraffin-embedded (FFPE) tissue specimens using the QIAamp DNA FFPE Advanced UNG Kit (Qiagen, Hilden, Germany). The DNA quantity will be determined using the dsDNA HS assay kit and the Qubit 2.0 Fluorometer (Invitrogen, Monza, Italy). Sequencing will be conducted using the Oncomine Precision Assay (Thermo Fisher Scientific, Waltham, MA, USA), which detects mutations, copy number alterations, and fusion transcripts in 50 genes, along with the Ion Torrent Genexus System. Data analysis will be performed using Torrent Suite Software 6.6 (Thermo Fisher Scientific). The limit of detection (LoD) for identifying genomic alterations with this assay is 5%.

Liquid biopsy is a non-invasive method for characterizing circulating tumor DNA released into the blood of cancer patients [23]. At least three phenomena associated with cancer contribute to enrich the bloodstream with tumor DNA: apoptosis, cellular necrosis, and active secretion. In cases with insufficient or inadequate tumor tissue, genomic profiling will be carried out on plasma samples. Specifically, circulating cell-free total nucleic acids (cfTNA) will be extracted from plasma samples using the MagMAX Cell-Free Total Nucleic Acid Isolation Kit (ThermoFisher Scientific) following the manufacturer’s instructions [24]. Sequencing will be performed using the Oncomine Pan-Cancer Cell-Free Assay, which detects mutations, copy number alterations, and fusion transcripts in 52 genes with a LoD of 0.1%, along with the Ion GeneStudio S5 System. Data analysis will be conducted using Torrent Suite Software v.5.18 (Thermo Fisher Scientific).

The identified variants from both tissue and plasma analyses will be verified using the Integrative Genome Viewer (IGV) from the Broad Institute, and mutations will also be annotated with ClinVar identifier numbers (https://www.ncbi.nlm.nih.gov/clinvar/intro/). This analysis will be conducted at the Cell Biology and Biotherapy Unit of the National Cancer Institute of Naples, “G. Pascale.“

### Analysis of T cell subgroups in the tumour microenvironment

Since the extent and quality of lymphocyte infiltration into tumor cores have been associated with outcomes in CRC, the presence of tumor-infiltrating T lymphocytes is crucial scientific data that will be recorded and correlated with other molecular data and clinical outcomes [25]. The analysis will be conducted through immunohistochemistry (IHC). Tissue sections of 4 μm FFPE tissues from primary and metastatic tumours will be immunostained using a biotin-streptavidin-peroxidase method. In particular, treatment with primary antibodies [CD3 anti-human, CD8 anti-human, FoxP3 anti-human, Granzyme B34 anti-human] will be followed by detection with a biotin/streptavidin-peroxidase/diaminobenzidine tetrahydrochloride-labeled secondary antibody. The immunostained slides will be acquired using the Aperio AT2 scanner. The resulting images will be analysed using dedicated software. This software will first highlight the areas where readings will be taken (peri-tumoral stroma, tumour margin, and tumour centre) and subsequently quantify lymphocytic biomarkers. Data obtained will be used for statistical analyses both as individual biomarkers and as a composite score of multiple markers, such as the formula that considers the numerical difference between GRZB + cells (cytotoxic cells) and FOXP3 + cells (immunosuppressive cells), on the total CD3 + cells. In addition, appropriate techniques of cellular and molecular biology will be used to characterize NK cells, Tregs, and chemokines in the primitive, metastatic, and peri-metastatic tissue. The analysis will be carried out at the Pathology and Cytopathology Unit of the National Cancer Institute of Naples, “G. Pascale”.

### Clinical scenarios and timing of molecular characterizations

**Fig. 1:**
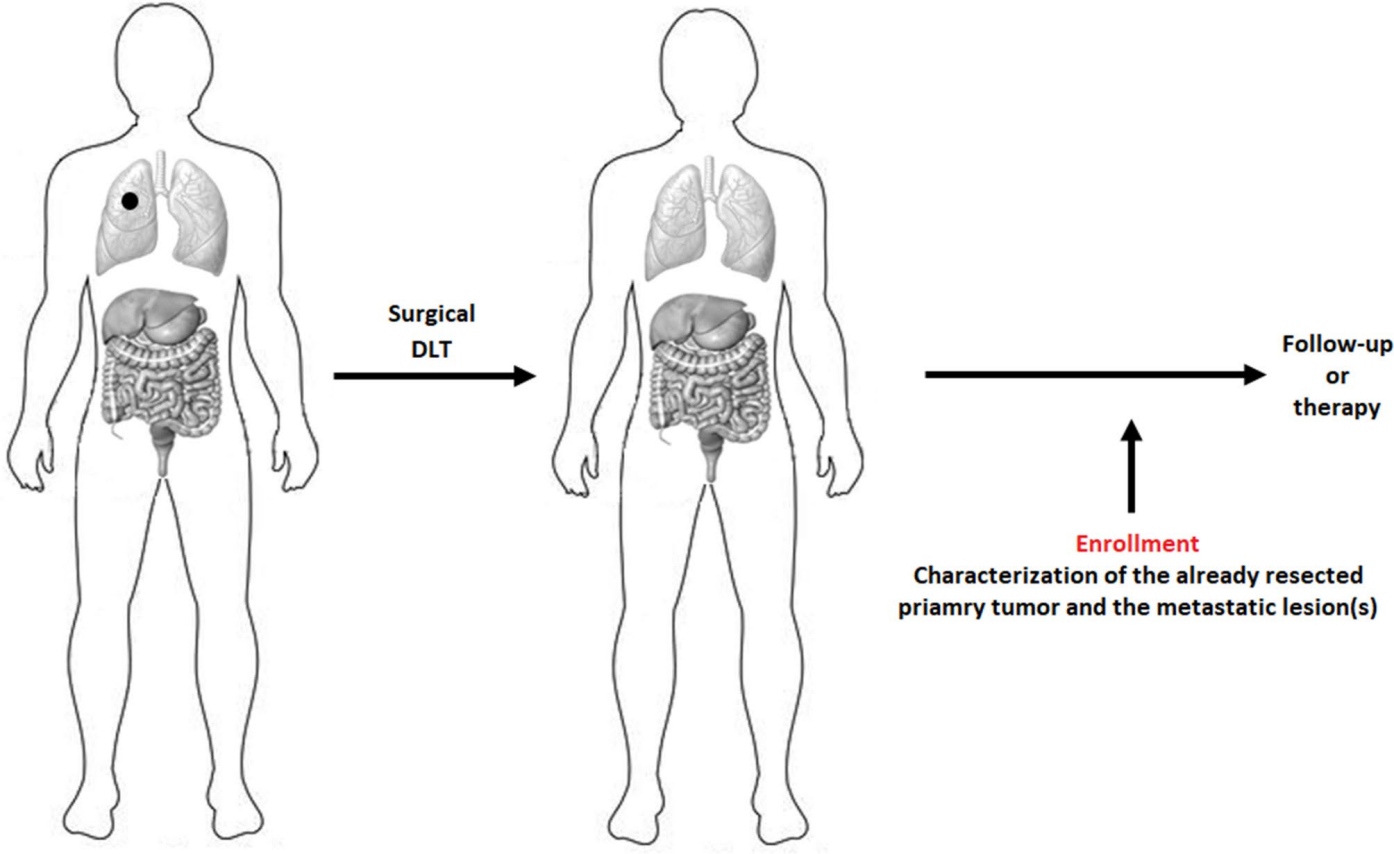
The removal of the primary tumor (in both “oligo-recurrence” or “sync-oligometastatic” disease presentation) is necessary for identifying genetic variations between it and any metastases. If synchronous or metachronous metastases are surgically excised, the samples collected during surgery will be used to perform molecular characterization. DLT: Definitive Local Therapy

**Fig. 2:**
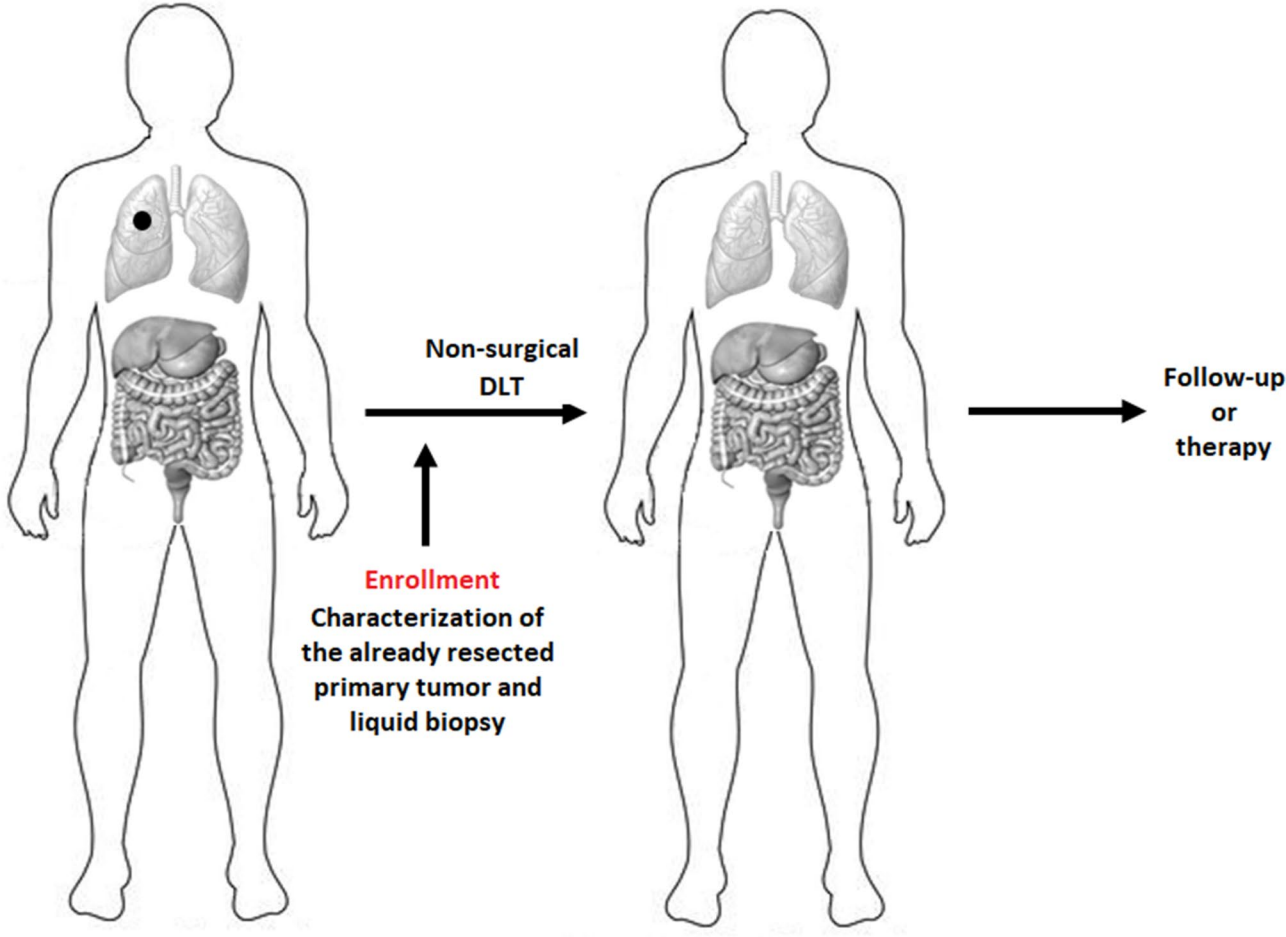
Liquid biopsies will be used to characterize oligometastatic lesions that are not suitable for upfront surgical removal. DLT: Definitive Local Therapy

The Figs. 1 and 2 represent the possible clinical scenarios of patients’ enrolment into the PREDICTION study. They are valid for both hepatic and pulmonary OMD.

### Sample storage times

FFPE samples will be stored for a period of 20 years. Blood samples, collected for routine hemato-biochemical evaluations, will be stored for 48 h after obtaining the results. Plasma samples intended for molecular analysis will be stored at -80 °C for a period of 10 years. The disposal of biological samples will be conducted in dedicated evaluation laboratories, and they will be treated as specialized medical waste.

### Response evaluation

The response will be evaluated based on the RECIST criteria (Response Evaluation Criteria in Solid Tumors) version 1.1, through Computed Tomography (CT) and/or Magnetic Resonance Imaging (MRI) according to clinical practice [17].

### Follow-up procedures

The follow-up procedures will follow the clinical practice. However, for patients enrolled in clinical trials, the procedures specified in each protocol will be followed. Follow-up procedures will begin in the presence of disease progression, which is defined as recurrence after radical surgery or progression according to the RECIST v1.1 criteria, from the time of enrolment in the study. For patients who do not progress, follow-up procedures will continue for at least one year.

### Ethical considerations

The study has received approval from the Ethics Committee of the Istituto Nazionale Tumori di Napoli, IRCCS “G. Pascale” (Approval No. 35/22). It will be conducted according to the protocol and procedures outlined in the Helsinki Declaration (1996), the Guidelines for Good Clinical Practice CPM/ICH135/95-DM 15/7/97, Legislative Decree no. 200 of 6 November 2007, Implementation of Directive 2005/2//EC Article 3, and GDPR EU Regulation no. 2016/679. The participants in the study will not be subject to any additional health risks or tests, nor is any harm to their physical integrity expected. To protect personal and clinical data, confidentiality will be maintained in accordance with privacy regulations. Participants will sign a specific informed consent form for the study, allowing them to exercise their right to information and self-determination regarding participation in the study. Patients may withdraw from the study at any time without providing any explanation. In such cases, related data will not be used in any analysis unless such analysis has already been completed before the patient’s withdrawal. Only data collected up to that point will be retained for research purposes.

## Discussion

It is important to reiterate that the PREDICTION study has two primary endpoints. This is a peculiarity driven by the scarcity of data in the scientific literature regarding the distinguishing characteristics between OMD and PMD. The first endpoint is descriptive in nature and has an exploratory spirit, aiming to determine the incidence of certain biological characteristics in various gastrointestinal tumors. The second endpoint is analytical and seeks to ascertain the impact of a molecular and immunological score in predicting the indolent behavior of OMD in colorectal cancer. This score has been constructed based on a previously described scientific rationale. It primarily relies on dynamic genetic features, specifically the comparison between the primary and metastatic tumor.

Cancer is an evolving genetic disease, and regrettably, defining the primary tumor genetics alone is insufficient, especially in colorectal cancer, for uniquely identifying OMD. As we have seen earlier, data from our group and others highlight genetic plasticity and how clones with driver mutations can vary over time. This dynamic and evolutionary phenomenon correlates with the oligo-metastatic behavior.

The PREDICTION study is the first prospective study proposing the identification of true OMD. The results could have significant implications for therapeutic strategies and clinical monitoring, enabling personalized and more intensive monitoring and treatment approaches in patients with a potentially ‘false’ oligo-metastatic status.

## Data Availability

Not applicable.

## Abbreviations

CD3: Cluster of differentiation 3
cfTNA: Cell-free total nucleic acids
CRC: Colorectal cancer
CT: Computed tomography
DLT: Definitive local therapy
ERBB2: Erythroblastic oncogene B-2
FFPE: Formalin fixed paraffin embedded
FOXP3: Forkhead box P3
GRZB: Granzyme B
IGV: Integrative genome viewer
IHC: Immunohistochemistry
KRAS: Kirsten rat sarcoma
MRI: Magnetic resonance imaging
NK: Natural killer
OMD: Oligometastatic disease
PIK3CA: Phosphatidylinositol-4,5-bisphosphate 3-kinase catalytic subunit alpha
PMD: Polymetastatic disease
PT: Primary tumor
RECIST: Response evaluation criteria in solid tumors
Tregs: T regulatory cells
